# Blood-Based α-Synuclein Biomarkers in Parkinson’s Disease: Molecular Diversity, Analytical Advances and Clinical Translation

**DOI:** 10.3390/ijms27146254

**Published:** 2026-07-14

**Authors:** Kailiang Ti, Yi Zhao, Eng King Tan, Dongrui Ma

**Affiliations:** 1 Department of Neurology, Singapore General Hospital, Singapore 169608, Singapore; ti.kai.liang@sgh.com.sg; 2 Division of Pathology, Singapore General Hospital, Singapore 169608, Singapore; zhao.yi@sgh.com.sg; 3 Department of Neurology, National Neuroscience Institute, Singapore 308433, Singapore; tan.eng.king@singhealth.com.sg

**Keywords:** PD, α-Syn, blood biomarkers, SAA, erythrocytes, biomarker standardization

## Abstract

Early and accurate diagnosis of Parkinson’s disease (PD) remains challenging in routine practice, as cardinal motor symptoms emerge only after substantial neurodegeneration has occurred. Blood-based biomarkers are therefore of considerable interest for identifying prodromal disease, enabling biologically stratified clinical trials, and supporting longitudinal monitoring. Among candidate markers, α-synuclein (α-Syn) is centrally relevant due to its fundamental role in PD pathogenesis. However, its peripheral measurement is complicated by marked molecular heterogeneity, uneven compartmental distribution, and analytical variability. In this narrative review, we examine the current evidence on blood-based α-Syn biomarkers with a focus on molecular diversity, compartmental biology, analytical platforms, and mechanisms governing central-peripheral exchange. We critically compare plasma, erythrocytes, and extracellular vesicle-based measurements and discuss the diagnostic implications of total, oligomeric, phosphorylated, and seeding-competent species. Recent advances in immunoassays, ultrasensitive technologies, mass spectrometry, and seed amplification assays (SAA) are also reviewed. Current evidence suggests that blood-based α-Syn assays hold promise, but their interpretation remains constrained by hemolysis, compartment-specific biology, assay heterogeneity, and limited longitudinal validation. Critically, no single blood-based α-Syn assay currently meets the performance standards required for standalone clinical application. Future progress will depend on methodological harmonization, multi-center and multi-ethnic studies, and integration with complementary biomarkers. A mechanistically grounded understanding of peripheral α-Syn dynamics is essential for developing clinically useful biomarker strategies for PD.

## 1. Introduction

PD is the second most common neurodegenerative disorder worldwide and is neuropathologically characterized by the progressive accumulation of misfolded α-Syn in Lewy bodies and Lewy neurites. Despite substantial advances in molecular understanding, diagnosis in routine practice remains primarily symptom-driven. The diagnosis is typically made only after the emergence of cardinal motor features (bradykinesia, rigidity, tremor, and postural instability), by which time substantial nigrostriatal degeneration—estimated at 50–70% of dopaminergic neurons—has already occurred [1]. This diagnostic delay poses major implications for both clinical care and therapeutic development, as potential disease-modifying interventions are likely to be most effective before widespread neuronal loss.

The clinical need for improved biomarkers is underscored by the limitations of current diagnostic approaches. The Movement Disorder Society (MDS) clinical diagnostic criteria for PD [2] achieve reasonable accuracy in specialized centers but remain imperfect, particularly in early stages. Misdiagnosis rates are substantial: approximately 10–20% of patients diagnosed with PD in early stages are later found to have alternative conditions, including multiple system atrophy (MSA), progressive supranuclear palsy (PSP), drug-induced parkinsonism, or essential tremor [3]. In community settings, diagnostic accuracy may be as low as 74–80% even when assessed by movement disorder specialists [4,5]. Furthermore, the distinction between PD and atypical parkinsonian syndromes (MSA, PSP, corticobasal degeneration) remains challenging, as overlapping clinical features and the absence of definitive molecular markers complicate differential diagnosis, particularly in the first 3–5 years after symptom onset. These limitations have intensified interest in biomarkers that more directly reflect underlying α-Syn pathology and can be applied in early-stage, prodromal, and longitudinal study settings [6].

The growing recognition that pathological α-Syn aggregation begins years—likely decades—before motor symptom onset has intensified efforts to develop biomarkers capable of detecting prodromal PD. Cerebrospinal fluid (CSF)-based assays, particularly SAA, have demonstrated strong diagnostic accuracy and support the concept that seeding competent α-Syn can be detected early in the disease course [7]. However, the invasiveness of the lumbar puncture procedure for obtaining CSF limits large-scale screening and longitudinal monitoring [8], underscoring the need for accessible peripheral alternatives such as blood-based assays.

Peripheral detection of α-Syn presents distinct biological and analytical challenges. Unlike CSF, blood contains multiple cellular and acellular compartments in which α-Syn is differentially distributed. Erythrocytes harbor the majority of circulating α-Syn [9], while plasma, extracellular vesicles (EVs), and lipoprotein-associated fractions contribute additional complexity. Furthermore, α-Syn exists in a spectrum of molecular forms, including monomeric, oligomeric, post-translationally modified, and strain-specific assemblies, each with distinct pathological relevance [10]. Pre-analytical variables, hemolysis, and methodological heterogeneity have historically contributed to inconsistent findings across studies.

Recent technological advances have reshaped this landscape. Ultra-sensitive immunoassays and mass spectrometry-based proteomics have significantly increased detection and quantification of α-Syn species, while SAAs can detect misfolded conformers through their aggregation-seeding activity, offering a functional measure more directly related to disease pathology. At the same time, emerging insights into blood–brain barrier transport, lipoprotein interactions, EV trafficking, and peripheral clearance mechanisms suggest that circulating α-Syn reflects a dynamic equilibrium between central production and systemic handling rather than a passive spillover phenomenon.

In this narrative review, we integrate molecular biology, compartmental distribution, analytical methods, and transport mechanisms to present a cohesive framework for understanding blood-based α-Syn biomarkers in PD. We examine how structural heterogeneity and post-translational modifications affect diagnostic interpretation, critically evaluate plasma and erythrocytic measurements, and highlight the transformative role and current limitations of SAA. Finally, we contextualize peripheral α-Syn within the broader biological pathways governing central-peripheral exchange and clearance. Rather than viewing peripheral α-Syn as a passive surrogate of central pathology, we propose that circulating species reflect a dynamic equilibrium shaped by aggregation biology, compartmental sequestration, and clearance pathways. Clarifying this equilibrium is essential for translating peripheral detection into clinically meaningful diagnostics.

## 2. Literature Search Strategy and Methods

This is a narrative review designed to provide a comprehensive and critical synthesis of the current literature on blood-based α-Syn biomarkers in PD. Given the broad and rapidly evolving nature of the field, a narrative approach was chosen to integrate findings across molecular biology, analytical chemistry, and clinical translation.

Search Strategy: We conducted systematic literature searches in PubMed and Web of Science for articles published from January 2000 to June 2026. The following search terms were used in various combinations: (“alpha-synuclein” OR “α-synuclein”) AND (“blood” OR “plasma” OR “serum” OR “erythrocyte” OR “red blood cell” OR “extracellular vesicle” OR “exosome”) AND (“biomarker” OR “diagnosis” OR “Parkinson’s disease”). Additional searches were performed using (“seed amplification assay” OR “RT-QuIC” OR “PMCA” OR “SAA”) AND (“blood” OR “plasma” OR “serum”).

Inclusion Criteria: We included original research articles, meta-analyses, and systematic reviews published in English that reported on blood-based α-Syn measurements in human participants with PD or prodromal populations. Preclinical studies, animal model studies, and studies focused exclusively on CSF or tissue biomarkers were included only when they provided mechanistic insight relevant to peripheral α-Syn biology.

Study Selection and Synthesis: Titles and abstracts were screened for relevance, and full texts were retrieved for detailed evaluation. Given the narrative nature of this review, we prioritized studies with robust methodologies, large sample sizes, and those addressing key controversies or knowledge gaps. Reference lists of included articles were also hand-searched to identify additional relevant publications. The information synthesized in this review is organized thematically around molecular diversity, compartmental distribution, analytical platforms, transport mechanisms, and clinical translation.

Limitations of the Review: As a narrative review, this article does not provide a quantitative meta-analysis of diagnostic performance metrics. Instead, we aim to provide a conceptual framework and critical appraisal of the field, identifying both promising avenues and persistent challenges.

## 3. Molecular and Structural Diversity of α-Synuclein

α-Syn is a 140-amino acid intrinsically disordered protein abundantly expressed in neurons and, to a lesser extent, in peripheral tissues including erythrocytes. Its conformational plasticity underlies both its physiological function and pathological potential. For biomarker development, understanding the molecular heterogeneity of α-Syn is essential, as distinct structural states and post-translational modifications may differentially reflect disease burden, propagation capacity, and diagnostic performance.

### 3.1. Conformational States and Aggregation Pathways

Under physiological conditions, α-Syn predominantly exists as a soluble monomer with a dynamic, unstructured conformation in solution. Upon interaction with lipid membranes, particularly those enriched in acidic phospholipids, the N-terminal domain adopts an α-helical structure that facilitates membrane binding and synaptic vesicle regulation [11]. However, under conditions favoring misfolding such as oxidative stress, altered lipid composition, or increased local concentration, α-Syn undergoes conformational transition toward β-sheet-rich assemblies [12].

The aggregation pathway is generally conceptualized as a multistep process involving monomers, soluble oligomers, protofibrils, and mature fibrils [12]. Among these species, soluble oligomers are widely considered to be the most cytotoxic forms, capable of membrane permeabilization, mitochondrial dysfunction, and synaptic impairment [12]. Mature fibrils, in contrast, are the principal constituents of Lewy bodies and Lewy neurites observed in PD pathology [13].

For peripheral biomarker development, distinguishing between total α-Syn and the relative abundance of aggregation-prone species is critical. Total protein levels may not accurately reflect pathogenic burden, particularly in early disease stages when misfolded species constitute only a minor fraction of the circulating pool. This distinction has important implications: an assay measuring total α-Syn may show no difference between PD and controls, while an oligomer-specific assay might demonstrate significant elevation.

### 3.2. Post-Translational Modifications and Pathogenic Significance

α-Syn undergoes multiple post-translational modifications (PTMs), including phosphorylation, truncation, nitration, and ubiquitination, each of which may alter aggregation propensity and toxicity. Among these, phosphorylation at serine 129 (pS129) is the most extensively studied and represents the predominant modified form within Lewy bodies.

Experimental evidence indicates that pS129-α-Syn is enriched in pathological inclusions compared to soluble cytosolic pools [14], suggesting a close association with disease-relevant aggregation processes. Phosphorylation may influence conformational stability, aggregation kinetics, and interactions with chaperones or degradation pathways. However, whether pS129 directly drives aggregation or represents a downstream consequence of fibrillization remains debated [15].

Truncated forms of α-Syn, particularly C-terminally truncated species, have also been implicated in enhanced aggregation and seeding activity [16]. Proteolytic cleavage can remove negatively charged residues in the C-terminal region that normally stabilize the soluble monomeric form, thereby promoting intermolecular interactions and accelerating fibril formation [17]. Oxidative modifications, including nitration and methionine oxidation, may further modulate oligomer formation and structural stability. Collectively, these PTMs generate a heterogeneous population of α-Syn species with distinct biochemical and pathological properties.

For peripheral detection, the selective measurement of modified forms such as pS129 may enhance disease specificity relative to total α-Syn quantification. However, low abundance and analytical challenges necessitate highly sensitive and well-validated assay platforms. Critically, pS129 antibodies vary significantly in their specificity and cross-reactivity, and positive results obtained with one antibody may not be reproducible with another [18].

### 3.3. Seeding Competence and Strain Heterogeneity

A defining feature of pathogenic α-Syn is its capacity for self-templated misfolding. Misfolded assemblies can act as seeds, inducing conformational conversion of native α-Syn into β-sheet-rich aggregates. This prion-like propagation underlies the progressive anatomical spread of pathology observed in PD and related synucleinopathies [19]. Short α-Syn fibrils of less than 50 nm have been shown to be the most efficient for seeding [20].

Notably, accumulating evidence supports the existence of conformationally distinct α-Syn “strains,” characterized by differences in fibril structure, aggregation kinetics, and biological behavior [21]. These strain-dependent properties may account for clinicopathological heterogeneity across synucleinopathies, including PD, multiple system atrophy (MSA), and dementia with Lewy bodies (DLB). The relative contribution of oligomers versus fibrils to toxicity and seeding remains debated: some studies suggest fibrils are more toxic due to seeding [22], while others argue oligomers are more diffusible and directly damaging. Regardless, in the context of SAA, strain heterogeneity manifests as distinct kinetic signatures, including variations in lag phase duration, growth rate, and maximal fluorescence intensity (see Section 6) [23].

### 3.4. Implications of Molecular Diversity for Peripheral Biomarker Development

The structural and biochemical diversity of α-Syn has direct implications for peripheral biomarker strategies. First, total protein quantification may be insufficient to capture disease-relevant pathology, as pathogenic species represent only a subset of the circulating pool. Second, PTM-specific assays, such as those targeting pS129, may provide enhanced disease association but require careful validation to avoid cross-reactivity and low signal-to-noise ratios. Third, assays that measure functional properties such as seeding competence may more closely reflect the presence of biologically active misfolded conformers than static concentration-based measurements.

Taken together, the conformational plasticity, post-translational heterogeneity, and strain diversity of α-Syn highlight the need for biomarker approaches that move beyond simple abundance measurements. A mechanistically informed framework that distinguishes between molecular species and functional properties will be critical for advancing blood-based diagnostics in PD.

## 4. Peripheral Distribution of α-Synuclein in Blood

The development of blood-based biomarkers for PD requires a clear understanding of how α-Syn is distributed, compartmentalized, and biologically processed in peripheral blood. Circulating α-Syn is not a uniform entity but exists across multiple blood fractions, including plasma, erythrocytes, platelets, and extracellular vesicles. Among these, erythrocytes constitute the predominant reservoir, containing the vast majority (estimated > 95%) of measurable blood α-Syn [9]. This compartmental complexity has significant implications for assay interpretation, variability across studies, and diagnostic performance.

### 4.1. Plasma Versus Erythrocytes

Early investigations of blood α-Syn focused primarily on plasma or serum. However, high heterogeneity across cohorts rapidly emerged [24,25], largely attributable to pre-analytical confounders—most notably hemolysis. Because erythrocytes contain markedly higher concentrations of α-Syn than plasma [9], even minimal red blood cell lysis during sample collection or processing can artificially elevate plasma measurements. Differences in centrifugation protocols, freeze–thaw cycles, storage duration, and anticoagulant use further contribute to inter-study variability.

To mitigate these confounding effects, subsequent studies quantified α-Syn directly within erythrocytes. A brief analysis of available studies assessing blood α-Syn levels in PD patients (Section 4.5 and Table 1) [26,27,28,29,30,31,32,33,34,35,36,37,38,39,40,41,42,43] report increased total and pathogenic α-Syn levels in PD patients compared to healthy controls. These findings suggest that erythrocytic measurements may provide a more stable reflection of peripheral alterations than plasma-based assays.

Nevertheless, the comparative performance of plasma versus erythrocytic measurements is not universally consistent. In a study directly comparing plasma and erythrocytic α-Syn within the same cohort under standardized conditions, plasma measurements achieved a higher AUC than erythrocytic measurements [26], underscoring that compartmental superiority is not absolute and may depend on assay platform, disease stage, and pre-analytical rigor. Moreover, even within the erythrocyte compartment, different molecular species (total vs. oligomeric vs. phosphorylated) yield varying diagnostic performance. Rather than representing competing approaches, plasma and erythrocyte measurements may capture complementary aspects of α-Syn biology—plasma reflecting acute circulating levels and erythrocytes providing a time-integrated signal over the ~120-day lifespan of red blood cells.

Geographical variation further complicates interpretation. Studies conducted in certain East Asian populations have consistently reported elevated α-Syn levels across both plasma and erythrocytic compartments in PD patients, whereas findings from European and North American cohorts have been more heterogeneous (Table 1). If representative of broader populations, these discrepancies might reflect genetic, environmental, or methodological differences rather than universal biological effects. Large multi-ethnic validation cohorts and harmonized meta-analyses will be essential to disentangle these factors.

### 4.2. Biological Characteristics of Erythrocytes

The predominance of α-Syn within erythrocytes raises important biological questions. Although red blood cells are anucleate and lack organelles, they possess a highly specialized proteome dominated by hemoglobin. α-Syn is present in erythrocytes at concentrations orders of magnitude higher than those observed in plasma, suggesting either active incorporation during erythropoiesis or stable retention during maturation.

The erythrocyte intracellular environment is characterized by high oxidative flux due to continuous oxygen transport and iron-containing heme groups. Oxidative stress is known to influence α-Syn aggregation and conformational stability through nitration [44], potentially promoting oligomer formation or post-translational modifications. The lipid composition of the erythrocyte membrane, enriched in negatively charged phospholipids, may further modulate α-Syn membrane binding and aggregation propensity [45].

Human red blood cells circulate for approximately 120 days before clearance by the reticuloendothelial system. Consequently, erythrocytic α-Syn levels likely reflect a time-integrated signal, reflecting cumulative exposure rather than acute fluctuations. This temporal integration could theoretically enhance stability as a biomarker but may also blunt sensitivity to rapid disease progression or short-term therapeutic effects.

Despite growing interest in erythrocytic α-Syn as a stable peripheral biomarker, a fundamental biological question remains unresolved: whether erythrocytic α-Syn primarily reflects central nervous system pathology or represents an independent peripheral pool with distinct regulatory dynamics. Several non-mutually exclusive mechanisms may contribute (See Section 7 for detailed discussion). Longitudinal changes, rather than absolute concentrations, may more faithfully reflect shifts in systemic or central α-Syn dynamics, but this hypothesis requires direct experimental validation.

### 4.3. Molecular Forms in Circulation

Peripheral α-Syn exists in multiple molecular forms, including monomeric, oligomeric, phosphorylated, and protein-bound complexes. Most clinical studies have focused on total α-Syn and oligomeric species. Oligomer-specific assays have frequently demonstrated greater discriminatory power than total protein measurements [27,33,46,47], consistent with the pathogenic relevance of soluble oligomers.

Phosphorylated α-Syn at serine 129 (pS129) has received particular attention as a candidate peripheral biomarker [48]. Elevated erythrocytic pS129-α-Syn levels have been associated with disease duration and motor severity in several cohorts [41,43,49]. Differential expression across motor phenotypes suggests that phosphorylated species capture aspects of disease heterogeneity. Associations with non-motor features, including cognitive impairment and olfactory dysfunction, further support this possibility.

However, cohort sizes remain modest and longitudinal validation is limited. Variability in assay specificity and cross-reactivity for phosphorylated epitopes also complicates interpretation. Thus, while modified species such as pS129 may enhance disease association, their incremental diagnostic value over total or oligomeric measurements remains to be fully established in large, independent cohorts.

Beyond soluble forms, α-Syn may circulate bound to hemoglobin or associated with lipoproteins and extracellular vesicles. Hemoglobin-α-Syn complexes identified in erythrocytes raise the possibility of structured protein–protein interactions rather than passive coexistence [50]. Such complexes have also been found to be increased in PD patients , and may influence stability, detectability, and aggregation behavior [51,52].

### 4.4. EVs as a Specialized Compartment

EVs, including exosomes and microvesicles, represent another important vehicle for α-Syn transport. Neuron-derived EVs can carry α-Syn species into peripheral circulation, providing a potential mechanism for CNS-derived biomarker detection. EV-associated α-Syn may be enriched in aggregation-prone or seeding-competent forms relative to soluble plasma fractions.

Isolation of neuron-derived EVs from blood has been attempted using surface markers such as L1CAM [53]. However, the specificity of such markers has been questioned, with some studies suggesting that L1CAM may not exclusively label neuronal vesicles in plasma as they are widely expressed outside the brain and may also exist as soluble forms unattached to vesicles [54,55]. Methodological differences in ultracentrifugation, size-exclusion chromatography, and immunoaffinity capture further contribute to variability in EV yield and purity.

Functionally, EV-associated α-Syn has been shown to retain seeding competence in vitro, and in some studies, blood-derived EV fractions have demonstrated improved SAA sensitivity compared with whole plasma [55]. However, technical complexity, cost, and reproducibility concerns currently limit widespread clinical application. Standardized EV isolation protocols and validation of cell-type-specific markers remain priorities for the field.

### 4.5. Diagnostic Performance and Cohort Variability

Most available evidence supports increased oligomeric and total α-Syn in blood compartments of PD patients relative to controls. However, diagnostic sensitivity and specificity vary substantially across studies and are generally insufficient for standalone clinical implementation when based solely on concentration measurements.

Table 1 provides a summary of key studies evaluating blood α-Syn measurements in PD, with particular attention to methodological characteristics and diagnostic performance (A comprehensive version of Table 1 is provided in the Supplementary Table S1). Several critical observations emerge from this synthesis:

First, reported area-under-the-curve (AUC) values for plasma and erythrocytic measurements typically range between approximately 0.65 and 0.85 (Table 1), reflecting moderate discrimination with substantial inter-study variability. These values are insufficient for standalone diagnostic use, where AUC > 0.90 would generally be expected for a screening or diagnostic test.

Second, conflicting findings are common. While many studies report elevated α-Syn in PD, others have found no significant difference or even decreased levels [25,28]. These discrepancies likely reflect pre-analytical differences, assay heterogeneity, and population-specific factors. For example, two studies using the same commercial ELISA kit in different populations reported opposite directions of effect [26,30], highlighting the influence of cohort-specific factors or unmeasured confounders. Meta-analytic summaries of these heterogeneous findings are provided in Supplementary Table S2. A recent meta-analysis by Shu et al. further confirmed the diagnostic potential of peripheral α-Syn while highlighting substantial heterogeneity across studies [56].

Third, the lack of standardized reference materials and calibrators makes direct comparison across studies problematic. Even within the same assay platform, inter-laboratory differences in sample processing can produce divergent results.

Fourth, cross-sectional designs predominate in the literature, limiting conclusions regarding prognostic value. Whether peripheral α-Syn alterations precede motor onset, track longitudinal progression, or respond to therapeutic intervention remains incompletely characterized. Emerging data in prodromal populations—such as individuals with REM sleep behavior disorder—suggest potential early alterations [32], but robust prospective validation is needed.

Fifth, several studies have failed to differentiate PD from healthy controls using α-Syn levels alone [25,28], raising issues with biological and analytical variability. It is particularly noteworthy that the diagnostic performance of plasma and erythrocytic α-Syn rarely exceeds that of established clinical assessment in specialized centers.

Sixth, the sensitivity of blood-based measurements may be limited by the dilution effect of the large peripheral α-Syn pool. Given that erythrocytes contain orders of magnitude more α-Syn than plasma, subtle CNS-derived signals may be masked [9].

In summary, the peripheral distribution of α-Syn is biologically complex and compartment-dependent. While erythrocytes provide a relatively stable and abundant source for measurement, interpretation requires careful consideration of molecular form, pre-analytical variables, and cohort heterogeneity. These limitations underscore the need for analytically robust platforms and mechanistically informed assay design, as discussed in subsequent sections. Thus, peripheral α-Syn concentration measurements should not yet be considered a standalone diagnostic marker, but rather as one component of a broader multimodal assessment strategy.

**Table 1:** Summary of key studies on blood α-Syn measurements in PD. (Note: A comprehensive version of this table with detailed sensitivity/specificity values is provided in the Supplementary Table S1).

Refs.	Cohort Size (PD/HC)	Sample Type	Assay Platform	Key Finding	Reported Performance	Limitations
[27]	34/27	Plasma	ELISA (oligomer)	↑ Oligomeric α-Syn in PD	AUC ~0.74	Small cohort; validation lacking
[28]	30/40	Plasma	ELISA (total)	↓ Total α-Syn in PD	AUC ~0.67	Contradicts later studies; assay specificity unclear
[30]	57/57	Plasma	ELISA (total)	No significant difference	AUC ~0.55	Highlights heterogeneity
[26]	96/80	Plasma/RBC	ELISA	↑ α-Syn in both compartments; plasma performed better	AUC plasma 0.85; RBC 0.72	Single-center; Chinese cohort
[36]	62/52	RBC	ELISA (total/oligomer)	↑ Oligomeric α-Syn in PD	AUC 0.78	Small sample; no longitudinal data
[39]	147/132	RBC	ELISA (total/oligomer)	↑ Total and oligomeric α-Syn; correlated with disease severity	AUC 0.82	Cross-sectional; Chinese cohort
[40]	124/100	RBC	ELISA (pS129)	↑ pS129-α-Syn in PD; differentiated subtypes	AUC 0.79–0.85	Limited follow-up; single center
[42]	201/150	RBC	ELISA (multiple species)	Multiple species elevated; combination improved accuracy	AUC 0.86	Cross-sectional; validation needed
[43]	60/45	RBC	ELISA (aggregated)	↑ Aggregated α-Syn in PD	AUC 0.81	Greek cohort; modest size
[57]	99/82	Serum	SAA	Seeding activity in PD; distinguished from MSA	Sensitivity 89%; Specificity 92%	Japanese cohort; promising but preliminary
[58]	130/89	Plasma EVs	SAA	EV-enriched SAA improved detection	AUC 0.85	EV isolation complex; preliminary
[59]	89/55	Serum	SAA	Seeding activity detected in PD and prodromal	AUC 0.89	Serum SAA; requires validation

Abbreviations: PD, Parkinson’s disease; HC, healthy control; RBC, red blood cell; EV, extracellular vesicle; SAA, seed amplification assay; pS129, phosphorylated serine 129; AUC, area under the receiver operating characteristic curve; ↑ increased; ↓ decreased.

## 5. Analytical and Proteomic Approaches for Peripheral α-Synuclein Detection

**Figure 1:**
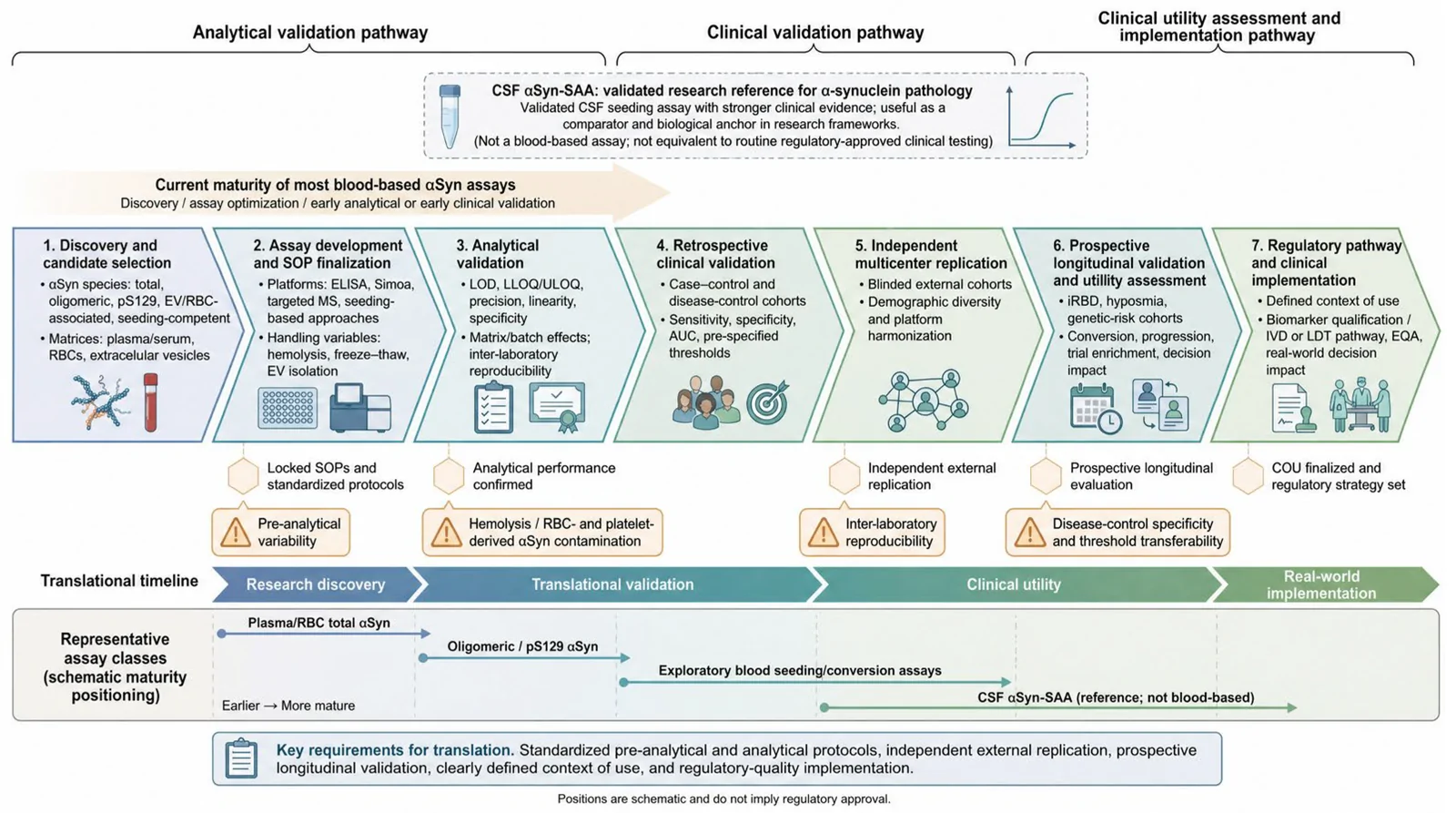
Developmental pipeline for blood-based α-Syn biomarkers. The translation of blood-based α-synuclein biomarkers from discovery to clinical practice requires a staged, iterative progression: candidate identification and assay development, analytical validation, retrospective and prospective clinical validation, demonstration of clinical utility, and eventual regulatory approval or real-world deployment. The shaded region highlights the approximate current maturity of most blood-based α-Syn approaches, which remain predominantly in the discovery, assay optimization, or early validation phases. CSF αSyn-SAA is shown separately as a more advanced reference framework and biological anchor, rather than as a blood-based comparator. Major translational bottlenecks include pre-analytical variability, hemolysis and erythrocyte contamination, heterogeneity in extracellular vesicle isolation, inter-laboratory reproducibility, disease-control specificity, and cut-off transferability. Successful clinical translation will depend on protocol standardization, independent external replication, prospective longitudinal validation, a clearly defined context of use, and implementation with regulatory-grade quality assurance. Created in BioRender. Zhou, Z. (2026) https://BioRender.com/0eek4yo, accessed on 9 July 2026.

The accurate quantification and characterization of peripheral α-Syn pose substantial challenges. Its heterogeneous molecular forms, low abundance in plasma, high abundance in erythrocytes, and susceptibility to pre-analytical variability necessitate carefully optimized and standardized methodologies. Differences in assay platforms and sample preparation protocols account for a significant proportion of inter-study variability reported in the literature. The developmental pipeline for blood-based α-synuclein biomarkers is summarized in Figure 1.

### 5.1. Sample Preparation and Extraction Strategies

Pre-analytical handling represents a critical determinant of assay reliability. As discussed in Section 4.1, hemolysis is a major confounder in plasma-based measurements [9]. Standardization of blood collection tubes, centrifugation protocols, storage temperature, and freeze–thaw cycles is therefore essential across all assay platforms.

For erythrocytic measurements, protein extraction presents additional complexity. The erythrocyte proteome is dominated by hemoglobin, which can obscure detection of lower-abundance proteins and modified α-Syn species [9]. Several extraction strategies have been employed to address this challenge. Chaotropic agents such as urea enable efficient protein denaturation and are compatible with downstream enzymatic digestion for proteomic workflows [60]. Detergent-assisted solubilization methods may enhance recovery of membrane-associated species but risk interference in subsequent immunoassays if not adequately removed [59]. Organic solvent precipitation [61] approaches can reduce lipid contamination but may compromise yield and reproducibility.

Hemoglobin depletion systems have been introduced to improve analytical sensitivity, particularly when targeting post-translationally modified or oligomeric forms of α-Syn. However, depletion efficiency and potential loss of protein complexes vary across commercial platforms. The lack of harmonized protocols complicates cross-cohort comparison and underscores the need for standardized extraction pipelines tailored specifically to erythrocytic α-Syn analysis.

### 5.2. Immunoassay Platforms and Ultra-Sensitive Technologies

Most early clinical studies relied on enzyme-linked immunosorbent assays (ELISA) to quantify total or oligomeric α-Syn. While widely accessible, conventional ELISA platforms are limited by relatively narrow dynamic range and susceptibility to antibody cross-reactivity. The detection of specific conformational states or post-translational modifications requires highly selective antibodies, and inter-batch variability can affect reproducibility.

Electrochemiluminescence (ECL) assays provide improved dynamic range and lower background noise compared to traditional ELISA [56]. More recently, single-molecule array (SIMOA) technology has enabled ultra-sensitive quantification of low-abundance proteins in plasma, reaching femtomolar detection limits [62]. Using SIMOA, Ng et al. demonstrated increased plasma α-Syn levels in PD patients [63]. These advances have expanded the feasibility of measuring peripheral α-Syn in early or prodromal disease stages, where concentrations may be modest.

Nevertheless, antibody specificity remains a critical limitation (see also Section 3.2 for discussion of pS129-specific antibodies). Distinguishing monomeric from oligomeric species requires rigorous validation against recombinant standards and aggregated controls, as epitope masking within fibrillar assemblies may confound immunodetection, Notably, several commercially available antibodies marketed as “oligomer-specific” have been shown to cross-react with monomers under certain conditions [18], highlighting the need for careful validation across all immunoassay.

### 5.3. Mass Spectrometry and Data-Independent Acquisition Approaches

Mass spectrometry-based proteomics offers complementary advantages by enabling direct peptide-level quantification and characterization of post-translational modifications. Immunoprecipitation coupled with liquid chromatography-tandem mass spectrometry (LC-MS/MS) can enrich α-Syn prior to analysis, improving specificity. However, data-dependent acquisition methods may exhibit limited reproducibility across laboratories due to stochastic sampling of low-abundance peptides.

Data-independent acquisition (DIA) platforms, including sequential window acquisition of all theoretical fragment ion spectra (SWATH-MS), address some of these limitations by systematically fragmenting all precursor ions within defined mass windows. This approach enhances reproducibility and facilitates quantitative comparison across cohorts. In erythrocytic samples, SWATH-MS has enabled detection of α-Syn peptides and associated protein complexes, including hemoglobin-α-Syn interactions [64].

Proteomic approaches also permit the detection of truncated or modified α-Syn species that may escape antibody-based assays. However, sensitivity for very low-abundance plasma species remains challenging without prior enrichment, and specialized instrumentation limits widespread clinical implementation.

### 5.4. Inter-Laboratory Variability and Standardization Challenges

Despite technological advances, inter-laboratory variability remains a major obstacle to clinical translation. Differences in sample handling, antibody selection, calibration standards, and analytical platforms contribute to inconsistent diagnostic performance across studies. Even within SAA, variation in recombinant substrate preparation, shaking conditions, buffer composition, and plate readers can influence kinetic parameters [65] (see Section 6.4 for a detailed discussion of SAA-specific challenges). Inter-laboratory reproducibility, though improving, remains imperfect.

Establishing harmonized reference materials and external quality control programs will be essential for advancing peripheral α-Syn assays toward regulatory approval. Multi-center validation studies employing standardized protocols and blinded sample analysis are particularly needed. Without such harmonization, reported sensitivity and specificity metrics may reflect methodological idiosyncrasies rather than true biological differences.

Table 2 provides a comparative overview of the major analytical platforms, highlighting their respective strengths, limitations, and clinical applicability. Detailed quantitative performance metrics for each platform are provided in Supplementary Table S3.

**Figure 2:**
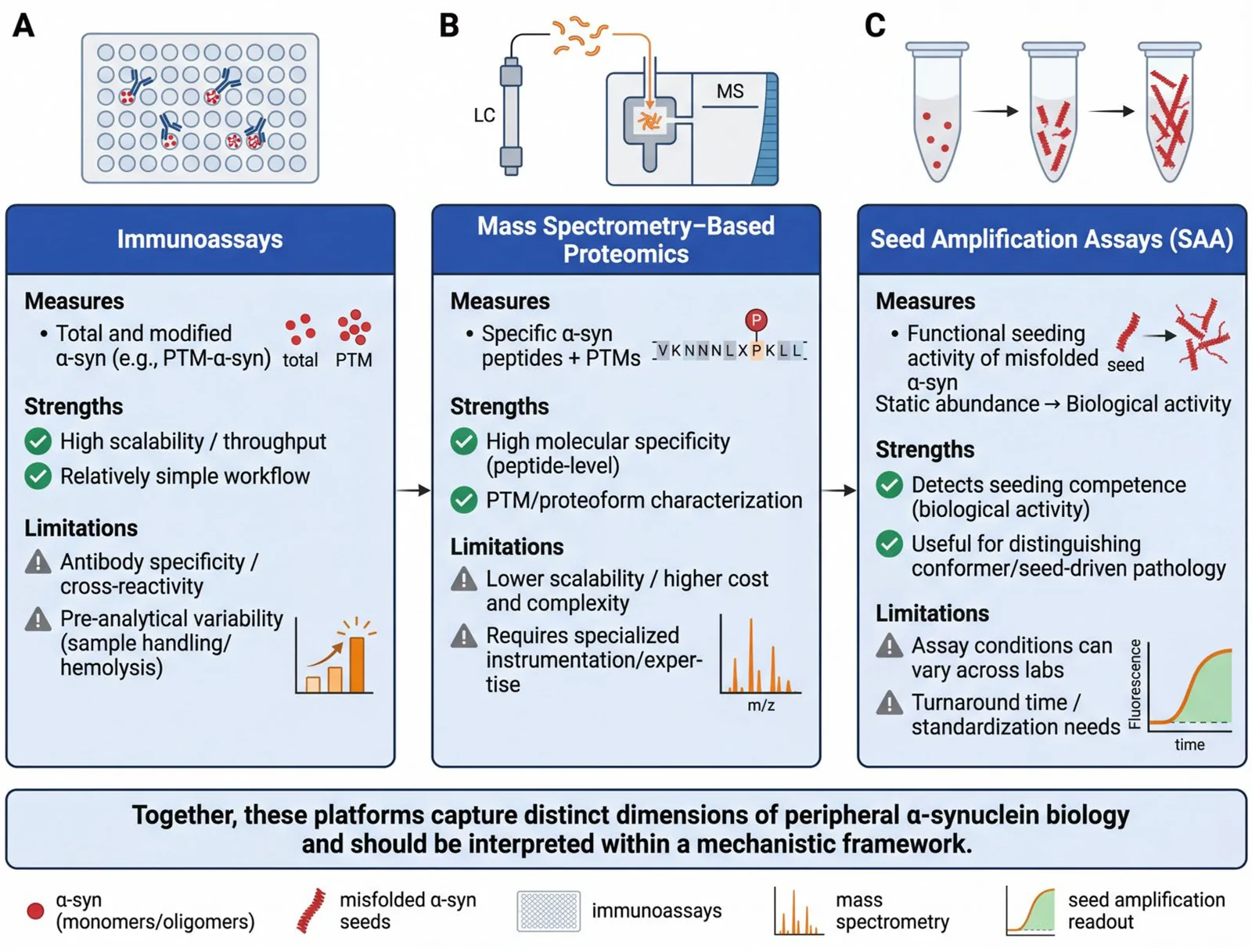
Comparative analytical platforms for blood-based α-Syn detection. Comparative overview of analytical platforms used for blood-based α-Syn detection. (**A**). Immunoassays primarily quantify total or modified α-Syn abundance and offer high scalability but are limited by antibody specificity and pre-analytical variability. (**B**). Mass spectrometry-based proteomics enables molecular characterization of specific peptides and post-translational modifications, providing enhanced specificity at the expense of scalability. (**C**). SAA interrogate the functional seeding competence of misfolded α-Syn, shifting biomarker assessment from static abundance to biological activity. Together, these platforms capture distinct dimensions of peripheral α-Syn biology and should be interpreted within a mechanistic framework. Created in BioRender. Zhou, Z. (2026) https://BioRender.com/fhfk0ia, accessed on 9 July 2026.

The conceptual differences among immunoassays, mass spectrometry-based proteomics, and seed amplification assays are summarized in Figure 2, highlighting that these platforms interrogate distinct biological dimensions of circulating α-Syn, ranging from protein abundance to functional seeding activity.

## 6. Seed Amplification Assays

While concentration-based measurements quantify the abundance of α-Syn in peripheral compartments, they do not directly assess the biological activity of misfolded species. SAA represent a conceptual and technical shift in biomarker development by detecting the seeding competence of pathological α-Syn conformers. By leveraging prion-like templating properties, these assays provide a functional readout that may more closely reflect disease-relevant pathology than static protein levels.

### 6.1. Principles of Seeding Amplification

SAA are based on the principle that misfolded α-Syn aggregates can template the conformational conversion of recombinant monomeric α-Syn into β-sheet-rich fibrils. In a typical assay, patient-derived samples containing putative seeds are incubated with excess recombinant substrate under agitation. Aggregation is monitored in real time using amyloid-sensitive fluorescent dyes, such as thioflavin T (ThT), which emit increased fluorescence upon binding to β-sheet structures [66].

The resulting kinetic curve typically comprises three phases: a lag phase corresponding to nucleation, an exponential growth phase reflecting fibril elongation, and a plateau phase indicating substrate depletion or reaction equilibrium [67]. Shorter lag phases and steeper growth rates are generally interpreted as indicative of higher seeding activity. However, kinetic parameters are influenced by assay conditions, including substrate concentration, buffer composition, temperature, and shaking intensity.

### 6.2. RT-QuIC and PMCA Methodologies

Two principal SAA platforms have been applied to α-Syn detection: real-time quaking-induced conversion (RT-QuIC) and protein misfolding cyclic amplification (PMCA). Both rely on repeated cycles of incubation and agitation to accelerate fibrillization, but they differ in shaking mechanics, reaction volumes, and kinetic monitoring strategies.

RT-QuIC assays typically employ intermittent shaking within multi-well plates, enabling real-time fluorescence monitoring across multiple samples [68]. PMCA, originally developed for prion protein detection, often involves more vigorous sonication cycles to enhance aggregate fragmentation and propagation [67]. In practice, methodological distinctions between these platforms have narrowed, and many contemporary α-Syn SAA incorporate elements of both approaches.

Critical variables include the source and purity of recombinant α-Syn substrate, buffer ionic strength, pH, and the presence of cofactors such as lipids. Substrate selection can influence assay sensitivity and strain discrimination capacity, underscoring the importance of protocol harmonization [65].

### 6.3. Diagnostic Performance in Cerebrospinal Fluid

SAA have demonstrated high diagnostic accuracy in CSF for distinguishing PD from healthy controls and other neurological disorders. Multiple independent cohorts report sensitivity and specificity frequently exceeding 85–90%, with some studies approaching near-complete separation between PD and controls [7,69,70,71]. However, a subset of clinically diagnosed PD patients remain SAA-negative; Fernandes Gomes et al. recently reported that such patients may exhibit Alzheimer’s disease-related traits [72]. Benchmark diagnostic performance data for CSF α-Syn-SAA from large multi-center cohorts are summarized in Supplementary Table S4.

Importantly, positive SAA results have been detected in prodromal populations, including individuals with REM sleep behavior disorder (RBD), years before motor symptom onset [7,73]. Patients with positive SAA results have also demonstrated a greater likelihood of phenoconversion to PD. These findings suggest that seeding-competent α-Syn aggregates may emerge early in the disease course and support the potential of SAA as a preclinical diagnostic tool.

Despite strong performance in CSF, translation to blood has been more challenging due to lower seed concentrations and increased biological complexity. Nevertheless, encouraging results from plasma and serum-based SAA studies indicate that blood-derived seeding activity can be detected with moderate-to-high diagnostic accuracy in selected cohorts.

### 6.4. Blood-Based SAA: Current Evidence and Limitations

Blood-based SAA extends the conceptual advantages of amplification assays to a more accessible biological fluid. Several groups have reported successful detection of seeding activity in plasma, serum, or blood-derived extracellular vesicles [57,58,74,75]. Diagnostic performance varies across studies but has reached AUC values consistent with clinically meaningful discrimination in some cohorts (Table 1).

Notably, blood-derived SAA may detect seeding activity even when total α-Syn concentrations are unchanged as it relies on the presence of pathogenic seeds. This suggests that functional aggregation capacity may better reflects pathogenic burden than absolute protein abundance.

However, blood-based SAA faces several critical challenges:

First, non-specific interference from blood matrix components represents a major obstacle. Hemoglobin, lipoproteins, immunoglobulins, and other abundant serum proteins can inhibit the aggregation reaction or cause spurious ThT fluorescence [75,76]. Lipoproteins, particularly ApoE-containing particles, have been shown to inhibit α-Syn aggregation in a concentration-dependent manner [77,78], potentially reducing assay sensitivity. Strategies to mitigate these effects include sample dilution, detergent pre-treatment, immunodepletion of abundant proteins, and isolation of EV fractions prior to amplification [57,74].

Second, false positives due to matrix effects have been reported in some studies. Even healthy individuals can show background ThT fluorescence or false-positive seeding signals, particularly in serum samples with high lipid content [73]. Establishing robust threshold values and internal controls is essential to distinguish true seeding from non-specific signal.

Third, variability across laboratories remains greater than in CSF-based assays, reflecting challenges in standardizing sample preparation and mitigating background interference [65,79].

Fourth, whether detected seeding activity originates from the CNS or from peripheral sources remains unclear. While EV-based enrichment may enhance CNS specificity, current markers for neuron-derived EVs (e.g., L1CAM) are imperfect and subject to debate (see Section 4.4 for detailed discussion) [53,54].

Fifth, the clinical sensitivity of blood-based SAA appears lower than that of CSF SAA in most head-to-head comparisons [80], suggesting that seeding-competent species are either present at very low concentrations in blood or significantly masked by peripheral inhibitors.

Despite these limitations, blood-based SAA represents one of the most promising avenues for peripheral α-Syn detection, as it interrogates functional pathology rather than static concentration. Continued refinement, multi-center validation, and methodological harmonization will determine their ultimate role in blood-based diagnostics for PD.

### 6.5. Strain Discrimination and Kinetic Signatures

Beyond binary diagnostic classification, SAA offer the potential to distinguish between synucleinopathies based on strain-dependent conformational properties. Differences in lag phase duration, aggregation rate, and maximal fluorescence have been reported between PD and MSA [23,81]. Although absolute kinetic values vary depending on assay conditions, relative differences suggest that distinct structural strains underlie these disorders.

Transmission electron microscopy and biochemical characterization of amplified fibrils further support structural divergence between disease entities. In some studies, PD-derived seeds generate paired or bundled filament morphologies, whereas MSA-derived seeds produce distinct twisted or straight filaments [58]. These findings are consistent with the broader concept of strain heterogeneity introduced in Section 3.

However, strain discrimination remains method dependent and biologically complex. Differences in recombinant substrate, reaction conditions, and analytical thresholds can influence apparent kinetic signatures. Furthermore, the clinical utility of strain discrimination in blood samples remains uncertain, given the lower seed concentrations and higher background noise compared to CSF. It is also unclear whether the kinetic signatures observed in blood samples truly reflect the same conformational strains as those in the CNS, or whether peripheral processing alters the detected conformers.

Thus, while SAA provides a promising avenue for differential diagnosis, assay harmonization is essential before strain-based classification can be implemented clinically.

## 7. Transport and Peripheral Handling of α-Syn

The interpretation of peripheral α-Syn measurements requires an understanding of how this protein traffics between the central nervous system (CNS) and the periphery, how it is transported within circulation, and how it is ultimately cleared. We propose that circulating α-Syn reflects a composite signal integrating central efflux, peripheral cellular sources, carrier-mediated transport, and reticuloendothelial clearance. Elucidating these pathways is essential for contextualizing blood-based biomarkers and understanding their biological relevance. It should be emphasized, however, that many of the mechanisms discussed in this section remain incompletely characterized and are currently based on experimental evidence that may not fully recapitulate human physiology.

**Figure 3:**
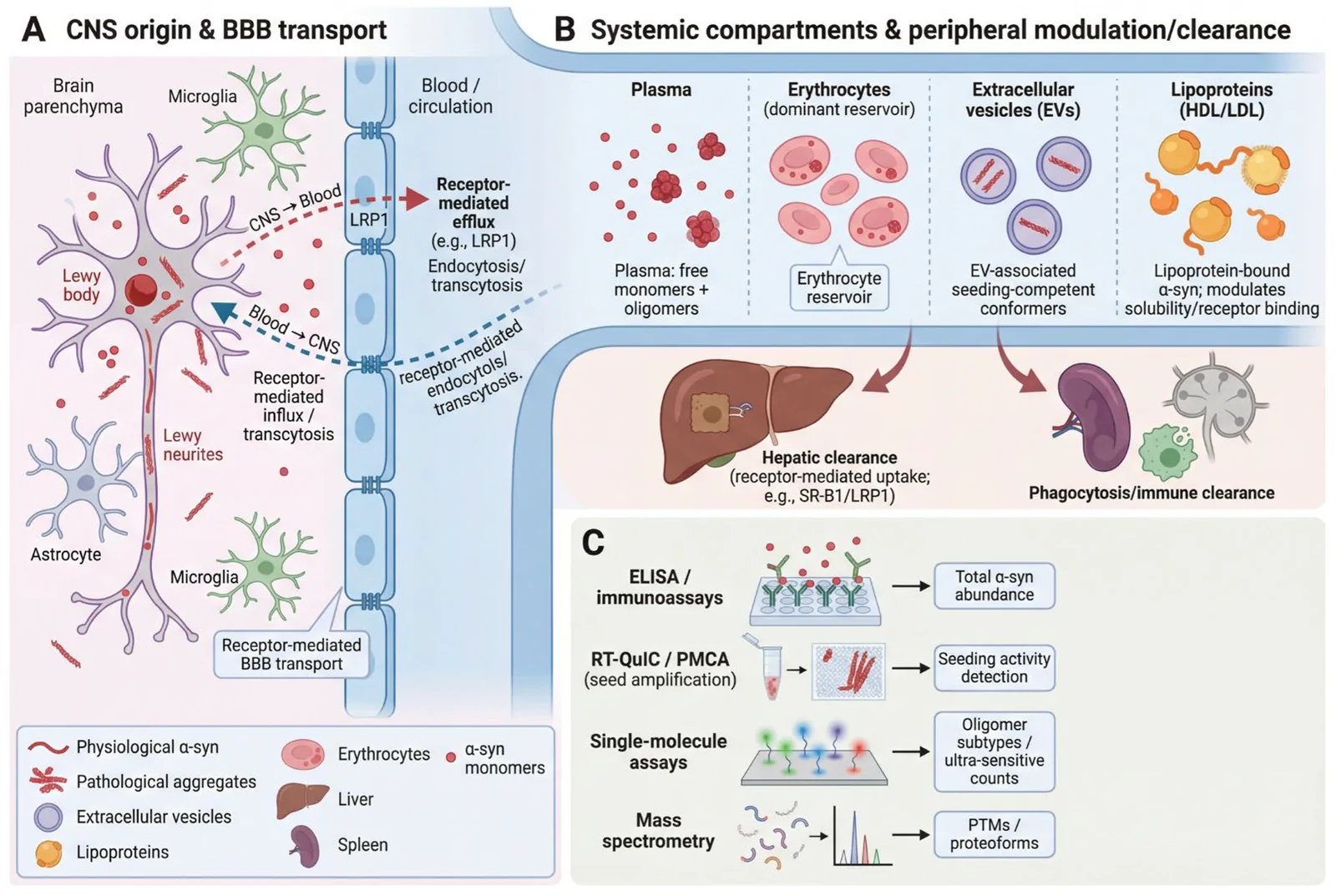
Central-peripheral transport and systemic handling of α-Syn in PD. Schematic illustration of central-peripheral α-Syn dynamics in PD. (**A**). Misfolded and seeding-competent α-Syn species arise within the CNS and may undergo bidirectional transport across the blood–brain barrier via receptor-mediated mechanisms. (**B**). In circulation, α-Syn exists in multiple compartments, including plasma, erythrocytes, lipoprotein-associated fractions, and extracellular vesicles. Erythrocytes represent the predominant peripheral reservoir, whereas extracellular vesicles may carry seeding-competent conformers. Lipoproteins and apolipoproteins modulate solubility and receptor interactions. (**C**). Peripheral clearance is mediated primarily by hepatic and reticuloendothelial pathways. Distinct analytical platforms interrogate different molecular aspects of circulating α-Syn, ranging from total protein abundance to functional seeding activity. Collectively, these interconnected processes define the systemic context within which blood-based measurements must be interpreted. Created in BioRender. Zhou, D. (2026) https://BioRender.com/4swj22d, accessed on 9 July 2026.

The interconnected central-peripheral transport and clearance pathways that shape circulating α-Syn profiles are schematically illustrated in Figure 3.

### 7.1. Blood–Brain Barrier Transport

Experimental studies support the bidirectional transport of α-Syn across the blood–brain barrier (BBB) [82,83]. Both monomeric and aggregated forms have been shown to cross endothelial layers in vitro and in animal models, although transport efficiency appears dependent on molecular size and conformational state. Smaller soluble species may traverse more readily, whereas larger fibrillar assemblies exhibit limited permeability unless BBB integrity is compromised.

Receptor-mediated mechanisms have been implicated in α-Syn trafficking. Low-density lipoprotein receptor-related protein 1 (LRP1), a multifunctional endocytic receptor expressed on abluminal brain endothelial cells [84], has been shown to facilitate uptake of α-Syn species [85]. Given its established role in amyloid-β transport, LRP1 represents a plausible mediator of CNS-periphery exchange for misfolded proteins. Other receptors, including members of the LDL receptor family and the receptor for advanced glycation end products (RAGE), may also participate, although their precise contributions remain incompletely characterized.

Importantly, BBB integrity itself is dynamic and influenced by aging, inflammation, and neurodegenerative pathology. Subtle disruption of tight junction proteins or altered endothelial transcytosis could modulate α-Syn efflux into the bloodstream. Thus, peripheral α-Syn levels may reflect, at least in part, BBB functional status in addition to central aggregate burden. Longitudinal studies correlating blood α-Syn with imaging markers of BBB permeability (such as dynamic contrast-enhanced MRI) would help clarify this relationship.

### 7.2. Lipoproteins and Apolipoproteins

Once in circulation, α-Syn does not exist solely as a free soluble protein. Interactions with lipoprotein particles and apolipoproteins may influence its stability, solubility, and clearance. Apolipoprotein E (ApoE), best known for its role in Alzheimer’s Disease, has also shown to colocalize with α-Syn in lipoprotein vesicles [83]. It also binds to lipid surfaces and its uptake into cells is mediated by receptors such as LRP1 [85]. Several studies have found it to be inhibitory to the aggregation of α-Syn in SAA of both CSF and blood [77,78].

Isoform-specific differences in ApoE structure (ε2, ε3, ε4) alter receptor binding affinity and lipidation status [86], potentially influencing misfolded protein handling. Although ApoE genotype is not a primary risk factor for PD in the same manner as in Alzheimer’s disease, it may modulate disease expression or progression through effects on protein aggregation and transport.

Apolipoprotein AI (ApoAI), the principal component of high-density lipoprotein (HDL), has also been implicated in peripheral protein transport. HDL particles possess anti-inflammatory and antioxidant properties and may facilitate sequestration or clearance of misfolded proteins. Reduced HDL levels have been associated with PD risk [87], which suggests that lipid metabolism influences peripheral α-Syn dynamics.

These interactions suggest that circulating α-Syn may be partially lipoprotein-bound, which could alter its detectability in immunoassays and influence aggregation behavior in SAA. Stratification by lipid profile or apolipoprotein genotype may therefore refine biomarker interpretation. However, these interactions remain incompletely characterized in humans, and their physiological significance for PD biomarker performance is not yet established.

### 7.3. Extracellular Vesicles

As introduced in Section 4.4, EVs represent an important vehicle for α-Syn transport. Neuron-derived EVs can carry α-Syn species into peripheral circulation, and EV-associated α-Syn has been shown to retain seeding competence in vitro [70]. This suggests that EVs may concentrate pathogenic conformers and protect them from peripheral degradation, potentially serving as a mechanism for CNS-to-periphery transfer. However, whether EV-associated α-Syn in blood reflects genuine CNS pathology or peripheral contamination remains debated [53,54] (see Section 4.4 for a detailed discussion of EV isolation and biomarker applications).

### 7.4. Hepatic and Reticuloendothelial Clearance

Peripheral clearance of circulating proteins is primarily mediated by the liver and reticuloendothelial system. Kupffer cells, the resident macrophages of the liver, play a central role in scavenging circulating aggregates and protein complexes. Consistent with this, striatal injection of α-Syn in mice results in its subsequent localization and degradation in the liver, supporting a role for hepatic clearance [88]. Receptors such as LRP1 and LDLR expressed on hepatocytes [89] may facilitate uptake of lipoprotein-bound or free α-Syn species.

Comparisons with amyloid-β clearance pathways in Alzheimer’s disease are informative. In that context, peripheral “sink” mechanisms have been proposed [90,91], whereby plasma amyloid-β is sequestered through binding to peripheral carrier proteins, reducing measurable concentration of soluble amyloid-β in circulation. This sequestration may also generate a concentration gradient that promotes efflux of amyloid-β from the CNS into peripheral circulation. Whether similar dynamics apply to α-Syn remains uncertain, but it highlights the need for caution when inferring central pathology from peripheral measurements alone.

Splenic macrophages and other components of the mononuclear phagocyte system may also participate in aggregate clearance [92]. Age-related decline in reticuloendothelial efficiency could theoretically alter circulating α-Syn levels independent of central pathology. Inflammatory states, metabolic syndrome, and liver dysfunction may likewise modulate peripheral handling. These potential confounders are rarely accounted for in biomarker studies but could significantly influence results.

### 7.5. Temporal and Disease-Stage Considerations

Transport and clearance dynamics likely evolve across disease stages. In early PD, low-level central aggregation may produce modest peripheral efflux detectable primarily through amplification-based assays. As disease progresses and aggregate burden increases, peripheral compartments may exhibit greater accumulation or altered distribution among molecular forms.

Conversely, advanced neurodegeneration with neuronal loss could reduce central α-Syn production, potentially influencing longitudinal biomarker trajectories. Distinguishing between central production, BBB permeability changes, and peripheral clearance efficiency will be essential for interpreting temporal patterns in blood-based measurements.

Critically, direct quantitative correlations between blood α-Syn species and neuropathological burden in humans remain extremely limited—few studies have performed post-mortem validation of blood α-Syn levels against brain pathology. This fundamental knowledge gap severely constrains our ability to interpret the biological significance of peripheral measurements.

In summary, peripheral α-Syn reflects a dynamic equilibrium between central efflux, lipoprotein-mediated transport, EV trafficking, and hepatic or reticuloendothelial clearance. Understanding these interconnected pathways provides a mechanistic framework for interpreting why different analytical platforms yield distinct peripheral signals. However, this framework remains largely conceptual and requires substantial experimental validation in humans. A transport-informed perspective offers a rational basis for contextualizing variability across cohorts and assay platforms, but direct evidence linking blood measurements to neuropathological burden is still lacking.

## 8. Clinical Translation and Future Directions

The ultimate value of peripheral α-Syn as a biomarker lies not in analytical sophistication alone, but in its clinical applicability. For a blood-based assay to meaningfully impact PD management, it must demonstrate reproducibility, stage sensitivity, scalability, and integration into existing diagnostic frameworks. Bridging the gap between experimental validation and clinical implementation requires careful consideration of screening strategies, trial design, combinatorial biomarker panels, and regulatory pathways.

### 8.1. Early and Prodromal Diagnosis

One of the most compelling applications of blood-based α-Syn detection is the identification of prodromal PD. Clinical diagnosis of PD currently relies on motor manifestations that emerge only after substantial dopaminergic neuronal loss. This diagnostic delay limits the feasibility of disease-modifying interventions.

Individuals with isolated REM sleep behavior disorder (iRBD), hyposmia, or subtle autonomic dysfunction represent enriched populations at elevated risk for conversion to overt synucleinopathy. In such cohorts, blood-based SAA have demonstrated the presence of seeding-competent α-Syn years before motor onset, albeit in only approximately 30% of the iRBD cohort in some studies [7]. A minimally invasive blood-based assay could therefore serve as a screening tool for high-risk populations, enabling early enrollment into neuroprotective trials.

However, ethical and practical considerations arise: psychological burden of pre-symptomatic diagnosis, variable conversion rates, and the lack of proven disease—modifying therapies to offer to screen—positive individuals [93,94]. The risk of developing PD with a positive screening test needs to be determined, as well as optimal cut-off thresholds. Longitudinal cohort studies with standardized follow-up will be essential to determine positive predictive value and optimal cut-offs.

In the general population, where PD prevalence is approximately 0.3% [3], even a test with 99% specificity would yield many false positives, making population-wide screening currently infeasible. Therefore, blood-based assays for screening are most appropriate in enriched populations (e.g., iRBD, hyposmia, genetic risk carriers).

### 8.2. Biomarker-Guided Clinical Trials

The failure of numerous disease-modifying trials in PD has underscored the need for biologically stratified study populations. Inclusion criteria based solely on clinical diagnosis may inadvertently enroll patients without active α-Syn pathology or at heterogeneous disease stages.

Peripheral α-Syn assays—particularly those assessing seeding activity—could serve as inclusion biomarkers to enrich for patients with active synucleinopathy. Furthermore, longitudinal monitoring of blood-based seeding activity or modified α-Syn species may provide pharmacodynamic readouts of therapeutic efficacy.

For example, therapies targeting α-Syn aggregation, immunotherapy approaches, or agents modulating lipid metabolism could be evaluated using peripheral assays as secondary endpoints [95]. Establishing whether changes in blood α-Syn correlate with clinical progression or imaging markers will be critical for validating such applications.

### 8.3. Multi-Biomarker Combination Strategies

It is unlikely that a single biomarker will fully capture the complexity of PD pathophysiology. Multimodal panels combining α-Syn assays with complementary markers may enhance diagnostic accuracy and disease stratification.

Neurofilament light chain (NfL), a marker of neuroaxonal injury, has demonstrated utility in diagnosing PD and differentiating PD from atypical parkinsonian syndromes [96,97,98]. Combining α-Syn SAA with plasma NfL measurements may improve both sensitivity and specificity for synucleinopathy. For differential diagnosis, the combination of a positive SAA (indicating synucleinopathy) with elevated NfL (indicating rapid axonal degeneration) could help distinguish PD from MSA or PSP, where NfL levels are generally higher [92].

Other complementary biomarkers are under active investigation: Glial fibrillary acidic protein (GFAP): emerging as a marker of astrogliosis in PD [99]; Lysosomal enzymes (e.g., GBA, cathepsin D): may reflect impaired protein clearance pathways [94]; Inflammatory markers (e.g., TNF-α, IL-6): may capture neuroinflammatory components [95]; Metabolomic signatures: may provide systemic metabolic insights [97]; Genetic risk markers (e.g., GBA, LRRK2 variants): may stratify patients by underlying pathogenesis.

For different disease stages, distinct biomarker combinations may be optimal: Prodromal screening: SAA + genetic risk + clinical risk factors (e.g., RBD, hyposmia); Early diagnosis: SAA + NfL + dopamine transporter (DaT) imaging; Differential diagnosis: SAA (for synucleinopathy) + NfL (for axonal injury) + imaging patterns; Progression monitoring: Oligomeric/pS129 α-Syn + NfL + clinical scales.

Machine learning approaches integrating clinical features, imaging data, and multi-analyte biomarker profiles could enable more precise classification of disease stage and subtype. However, large, well-characterized datasets will be required to train and validate such models [100].

### 8.4. Standardization and Regulatory Considerations

For peripheral α-Syn assays to transition from research tools to clinical diagnostics, rigorous standardization is imperative. This includes harmonized protocols for blood collection, processing, storage, and analysis. Reference standards, calibration materials, and inter-laboratory quality control programs must be established.

Regulatory approval will require demonstration of analytical validity, clinical validity, and clinical utility. Prospective, multi-center studies with predefined endpoints are necessary to confirm reproducibility and predictive performance. Cost-effectiveness analyses will also influence adoption for specific indications (screening, differential diagnosis, trial enrichment).

Importantly, assay scalability and turnaround time must align with clinical workflows (see Section 8.5 for detailed discussion of implementation barriers). While SAA offer high sensitivity, their multi-day incubation periods and technical complexity may limit immediate deployment, whereas simplified immunoassay formats may be more suitable for large-scale screening applications.

### 8.5. Real-World Implementation Barriers

Several practical barriers must be addressed before blood-based α-Syn assays enter routine clinical workflows:

1. Assay turnaround time: SAA typically require 2–5 days of incubation, incompatible with same-day results. Simplified or automated platforms (e.g., digital ELISA) could reduce turnaround.
2. Cost and infrastructure: SAA require specialised plate readers, shakers, and trained personnel. The cost per sample is substantially higher than conventional ELISA and may be prohibitive for large-scale screening.
3. Negative predictive value for screening: In the general population (PD prevalence ~0.3%), even a test with 99% specificity would yield many false positives. Thus, screening is only viable in enriched populations (e.g., iRBD, genetic carriers).
4. Harmonization: Without inter-laboratory standardization, results cannot be compared across sites. External quality assessment (EQA) schemes are urgently needed.
5. Sample availability and quality: Blood samples are generally accessible, but pre-analytical variability (hemolysis, freeze–thaw cycles, anticoagulant type) remains a major confounder that is difficult to control across different clinical settings.

### 8.6. Current Limitations and Knowledge Gaps

A critical appraisal of the field reveals several persistent limitations:

1. Small sample sizes: Most published studies include fewer than 200 participants per group, limiting generalizability and statistical power for subgroup analyses.
2. Lack of ethnic/racial diversity: The majority of studies have been conducted in East Asian and European populations. Findings may not generalize to other ethnic groups.
3. Cross-sectional design: Few studies have followed participants longitudinally to establish temporal relationships between biomarker changes and clinical progression.
4. Poor inter-laboratory reproducibility: Even with the same assay platform, results can vary substantially across different laboratories due to differences in sample handling, reagent batches, and data analysis pipelines.
5. No universally accepted reference standards: The absence of standardized recombinant α-Syn reference materials, calibrators, and quality controls makes cross-study comparison problematic.
6. Limited head-to-head comparisons: Few studies have directly compared multiple assay platforms (ELISA, SIMOA, MS, SAA) in the same cohort under standardized conditions.
7. Uncertain biological origin: The extent to which blood α-Syn reflects CNS pathology versus peripheral biology remains poorly characterized in humans.
8. Lack of post-mortem validation: Very few studies have correlated antemortem blood α-Syn measurements with post-mortem neuropathological findings.
9. Publication bias: Positive results are more likely to be published, potentially inflating the perceived diagnostic performance of blood α-Syn assays.

### 8.7. Future Directions

Several priorities emerge for future research:

First, longitudinal studies tracking peripheral α-Syn dynamics from prodromal to advanced stages are needed to define temporal trajectories and identify optimal windows for intervention.

Second, multi-ethnic cohorts should be included to address geographic variability observed in existing studies (Table 1).

Third, mechanistic investigations linking blood-based measurements with central pathology through imaging, CSF biomarkers, or post-mortem validation will strengthen biological interpretation.

Fourth, methodological harmonization through standardized protocols, reference materials, and EQA programs is essential.

Fifth, advances in detection technology—including microfluidics, digital immunoassays, and next-generation SAA formats—may further refine peripheral detection capabilities and reduce turnaround time.

Sixth, improved understanding of EV biology and lipoprotein interactions may reveal new avenues for assay development and enhance CNS specificity of blood-based measurements.

Seventh, integration of multi-omics approaches (proteomics, metabolomics, lipidomics) may identify novel biomarker panels that complement α-Syn measurements.

## 9. Conclusions

Parkinson’s disease is increasingly recognized as a disorder of misfolded protein accumulation and propagation long before the onset of overt motor symptoms. Yet clinical diagnosis remains anchored to late-stage manifestations, creating a critical gap between molecular pathology and therapeutic opportunity. Bridging this gap requires biomarkers that reflect the underlying biology of α-Syn rather than its downstream consequences.

Peripheral α-Syn detection has evolved from simple concentration-based measurements toward mechanistically informed interrogation of molecular form and biological activity. Structural heterogeneity, post-translational modifications, strain diversity, and compartmental distribution fundamentally shape diagnostic interpretation. SAA exemplify the paradigm shift by capturing seeding competence, while concentration-based approaches remain essential for contextual interpretation. Transport and clearance pathways—BBB transport, lipoprotein interactions, EV trafficking, hepatic clearance—define the systemic equilibrium that circulating α-Syn reflects.

Critically, the current evidence supports the promise of blood-based α-Syn assays, but substantial hurdles remain before clinical implementation. No single blood-based α-Syn assay currently meets the performance standards required for standalone application. Diagnostic AUC values for concentration-based assays typically range from 0.65 to 0.85, insufficient for confident clinical use. Blood-based SAA show more promise (AUC up to 0.89 in some cohorts), but require further validation and standardization. The field is characterized by substantial inter-laboratory variability, publication bias toward positive results, and a persistent lack of longitudinal validation.

We propose that the future of blood-based α-Syn biomarkers lies not in any single platform, but in harmonized integration of molecular specificity, analytical rigor, and mechanistic insight. With continued multi-center validation, longitudinal evaluation, and methodological standardization, peripheral α-Syn has the potential to transition from experimental promise to clinically actionable precision diagnostics in PD. However, this transition will require substantial investment in infrastructure, international collaboration, and regulatory frameworks to ensure that these assays deliver meaningful benefit to patients.

## Figures and Tables

**Table 2:** Comparative overview of analytical platforms for blood α-Syn detection.

Parameter	ELISA	SIMOA	Mass Spectrometry (DIA/SWATH)	Seed Amplification Assay (SAA)
Primary Target	Total/oligomeric/PTM α-Syn	Total/PTM α-Syn (ultrasensitive)	Peptide identification, PTM characterization	Functional seeding activity
Detection Limit	pg/mL–ng/mL	fg/mL–pg/mL	fmol–pmol range	Aggregation-based amplification
Throughput	High	Moderate	Low	Low-Moderate (multi-day assay)
Cost per Sample	Low-Moderate	Moderate-High	High	Moderate-High
Turnaround Time	Hours	Hours	Days	Days (2–5 days)
Equipment Required	Plate reader	SIMOA instrument	LC-MS/MS (high-end)	Fluorescence plate reader + shaker
Technical Complexity	Low	Moderate	High	High
Specificity	Antibody-dependent; variable	Antibody-dependent; improved	High (peptide-level)	High for misfolded conformers
Ability to Detect PTMs	Possible	Possible	Excellent (direct detection)	Indirect (seeding reflects conformation)
Standardization Level	Moderate	Moderate	Low-Moderate	Low (under development)
Major Strengths	Accessible; scalable; well-established	Ultrasensitive; good reproducibility	Unbiased; precise PTM detection	Functional readout; captures seeding
Major Limitations	Cross-reactivity; limited dynamic range	Cost; antibody availability	Instrumentation; sensitivity for low abundance	Standardization; time; false positives
Clinical Readiness	Moderate (research use)	Low-Moderate (emerging)	Low (research use)	Low-Moderate (CSF approved; blood in development)

Abbreviations: ELISA, enzyme-linked immunosorbent assay; SIMOA, single-molecule array; DIA, data-independent acquisition; SWATH, sequential window acquisition of all theoretical fragment ion spectra; PTM, post-translational modification; LC-MS/MS, liquid chromatography-tandem mass spectrometry.

## Data Availability

No new data were created or analyzed in this study. Data sharing is not applicable to this article.

## Supplementary Materials

The following supporting information can be downloaded at: https://www.mdpi.com/article/10.3390/ijms27146254/s1.
