# Correction: Ti et al. Blood-Based α-Synuclein Biomarkers in Parkinson’s Disease: Molecular Diversity, Analytical Advances and Clinical Translation. *Int. J. Mol. Sci.* 2026, *27*, 6254

**DOI:** 10.3390/ijms27188177

**Published:** 2026-09-14

**Authors:** Kailiang Ti, Yi Zhao, Eng King Tan, Dongrui Ma

**Affiliations:** 1 Department of Neurology, Singapore General Hospital, Singapore 169608, Singapore; ti.kai.liang@sgh.com.sg; 2 Division of Pathology, Singapore General Hospital, Singapore 169608, Singapore; zhao.yi@sgh.com.sg; 3 Department of Neurology, National Neuroscience Institute, Singapore 308433, Singapore; tan.eng.king@singhealth.com.sg

In the original publication [1], references 4, 15, 18, 68, 82, 83, 96, and 99 were incorrect. The correct references are provided below [2,3,4,5,6,7,8,9]. The authors state that the scientific conclusions are unaffected. This correction was approved by the Academic Editor. The original publication has also been updated.

## References

[1] Ti K., Zhao Y., Tan E.K., Ma D. (2026). Blood-Based α-Synuclein Biomarkers in Parkinson’s Disease: Molecular Diversity, Analytical Advances and Clinical Translation. Int. J. Mol. Sci..

[2] di Biase L., Pecoraro P.M., Di Lazzaro V. (2025). Validating the Accuracy of Parkinson’s Disease Clinical Diagnosis: A UK Brain Bank Case-Control Study. Ann. Neurol..

[3] Parra-Rivas L.A., Madhivanan K., Aulston B.D., Wang L., Prakashchand D.D., Boyer N.P., Saia-Cereda V.M., Branes-Guerrero K., Pizzo D.P., Bagchi P. (2023). Serine-129 phosphorylation of alpha-synuclein is an activity-dependent trigger for physiologic protein-protein interactions and synaptic function. Neuron.

[4] Vaikath N.N., Hmila I., Gupta V., Erskine D., Ingelsson M., El-Agnaf O.M.A. (2019). Antibodies against alpha-synuclein: Tools and therapies. J. Neurochem..

[5] Lashuel H.A., Surmeier D.J., Simuni T., Merchant K., Caughey B., Soto C., Fares M.B., Heym R.G., Melki R. (2025). Alpha-synuclein seed amplification assays: Data sharing, standardization needed for clinical use. Sci. Adv..

[6] Matsumoto J., Stewart T., Sheng L., Li N., Bullock K., Song N., Shi M., Banks W.A., Zhang J. (2017). Transmission of alpha-synuclein-containing erythrocyte-derived extracellular vesicles across the blood-brain barrier via adsorptive mediated transcytosis: Another mechanism for initiation and progression of Parkinson’s disease?. Acta Neuropathol. Commun..

[7] Paslawski W., Svenningsson P. (2023). Elevated ApoE, ApoJ and lipoprotein-bound alpha-synuclein levels in cerebrospinal fluid from Parkinson’s disease patients—Validation in the BioFIND cohort. Park. Relat. Disord..

[8] Lang A.E., Siderowf A., Macklin E.A., Poewe W., Brooks D.J., Fernandez H.H., Rascol O., Giladi N., Stocchi F., Tanner C.M. (2022). Trial of Cinpanemab in Early Parkinson’s Disease. N. Engl. J. Med..

[9] Tang Y., Han L., Li S., Hu T., Xu Z., Fan Y., Liang X., Yu H., Wu J., Wang J. (2023). Plasma GFAP in Parkinson’s disease with cognitive impairment and its potential to predict conversion to dementia. npj Park. Dis..

