# Supplementary material for: Blood-Based α-Synuclein Biomarkers in Parkinson’s Disease: Molecular Diversity, Analytical Advances and Clinical Translation
Source: Int J Mol Sci. 2026 Jul 14;27(14):6254. doi: 10.3390/ijms27146254 (PMC13410070; doi:10.3390/ijms27146254)
Supplement: Supplementary file 1 [file ijms-27-06254-s001.zip › ijms-4393420-supplementary.pdf]

Supplementary Materials

Supplementary Table S1. Comprehensive Summary of Blood-Based α-Synuclein Studies in Parkinson's Disease.

Reference	Cohort (PD/HC)	Sample Type	Molecular Target	Assay Platform	Key Findings	Diagnostic Performance	Blinded Assessment	Limitations / Bias
El-Agnaf et al. 2006 [1]	34 PD / 27 HC	Plasma	Oligomeric α-Syn	ELISA (oligomer-specific)	↑ Oligomeric α-Syn in PD vs. HC	Sensitivity 71%, Specificity 89% (AUC ~0.74)	Not reported	Small cohort; no independent validation
Li et al. 2007[2]	30 PD / 40 HC	Plasma	Total α-Syn	ELISA	↓ Total α-Syn in PD vs. HC	Sensitivity 63%, Specificity 70% (AUC ~0.67)	Not reported	Contradicts later studies; assay specificity unclear
Shi et al. 2010 [3]	57 PD / 57 HC	Plasma	Total α-Syn	ELISA	No significant difference between PD and HC	AUC ~0.55	Yes	Highlights heterogeneity; possible pre-analytical confounders
Duran et al. 2010 [4]	95 PD / 60 HC	Plasma	Total α-Syn	ELISA	↑ Total α-Syn in PD vs. HC; no difference between treated/untreated	Not reported	Not reported	No ROC analysis; cross-sectional
Wang et al. 2020 [5]	45 PD / 45 HC	Plasma & RBC	Total α-Syn	ELISA	↑ α-Syn in both compartments; plasma performed better	Plasma: Sens 80.0%, Spec 97.7% (AUC 0.85); RBC: Sens 71.1%, Spec 68.2% (AUC 0.72)	Not reported	Single-center; Chinese cohort; modest sample size
Zhao et al.	62 PD /	RBC	Total &	ELISA	↑ Oligomeric	AUC 0.78	Not	Small sample;

Reference	Cohort (PD/HC)	Sample Type	Molecular Target	Assay Platform	Key Findings	Diagnostic Performance	Blinded Assessment	Limitations / Bias
2016 [6]	52 HC		Oligomeric α-Syn	(oligomer-specific)	α-Syn in PD vs. HC		reported	no longitudinal data
Tian et al. 2019 [7]	147 PD / 132 HC	RBC	Total & Oligomeric α-Syn	ELISA	↑ Total and oligomeric α-Syn in PD; correlated with disease severity	AUC 0.82	Yes	Cross-sectional; Chinese cohort
Li et al. 2021 [8]	124 PD / 100 HC	RBC	pS129 α-Syn	ELISA (pS129-specific)	↑ pS129-α-Syn in PD; differentiated motor subtypes	AUC 0.79–0.85	Not reported	Limited follow-up; single center
Liu et al. 2022 [9]	82 PD / 65 HC	RBC	Total & Oligomeric α-Syn	ELISA	Longitudinal: levels stable over 3 years; no correlation with progression	Not reported	Yes	Modest sample; single center
Yu et al. 2022 [10]	201 PD / 150 HC	RBC	Total, Oligomeric, pS129 α-Syn	ELISA (multiple species)	Multiple species elevated; combination improved accuracy	AUC 0.86	Yes	Cross-sectional; validation needed
Dimoula et al. 2025 [11]	60 PD / 45 HC	RBC	Aggregated α-Syn	ELISA (aggregate-specific)	↑ Aggregated α-Syn in PD vs. HC	AUC 0.81	Not reported	Greek cohort; modest size
Chang et al. 2020 [12]	88 PD / 40 HC	Plasma & Serum	Total α-Syn	IMR immunoassay	↑ α-Syn in PD vs. HC	Plasma: AUC 0.92; Serum: AUC 0.99	Not reported	Very high AUC suggests possible overfitting; requires validation
Ng et al. 2019 [13]	170 PD / 51 HC	Plasma	Total α-Syn	SIMOA (Quanterix)	↑ Total α-Syn in PD vs. HC; higher levels in advanced	Not reported	Not reported	Commercial assay; antibody details not

Reference	Cohort (PD/HC)	Sample Type	Molecular Target	Assay Platform	Key Findings	Diagnostic Performance	Blinded Assessment	Limitations / Bias
					disease			disclosed
Foulds et al. 2011 [14]	189 PD / 91 HC	Plasma	Total & pS129 α-Syn	ELISA	pS129 α-Syn ↑ in PD vs. HC at baseline; total α-Syn trend (p=0.058)	Not reported	Not reported	pS129 elevation promising but assay specificity concerns
Cristiani et al. 2024 [15]	19 PD / 18 HC / 8 PSP	RBC	Total, Oligomeric, pS129 α-Syn	ELISA	RBC total α-Syn higher in PSP vs. PD and HC; distinguishes PSP from PD	AUC 0.853 (PSP vs. PD); Sens 100%, Spec 70.6%	Not reported	Pilot study; very small PSP sample (n=8)
Kluge et al. 2024 [16]	89 PD / 55 HC / prodromal	Serum	Seeding-competent α-Syn	SAA (RT-QuIC)	Seeding activity detected in PD and prodromal (iRBD)	AUC 0.89	Yes	Promising but requires multi-center validation
Okuzumi et al. 2023 [17]	99 PD / 82 HC / 30 MSA	Serum	Seeding-competent α-Syn	SAA (RT-QuIC)	Seeding activity in PD; distinguished from MSA	Sens 89%, Spec 92%	Yes	Japanese cohort; promising but preliminary
Schaeffer et al. 2024 [18]	130 PD / 89 HC	Plasma (EV-enriched)	Seeding-competent α-Syn	SAA (PMCA)	EV-enriched SAA improved detection vs. whole plasma	AUC 0.85	Not reported	EV isolation complex; technical variability
Wang et al. 2015 [19]	40 PD / 40 HC	RBC	Oligomeric α-Syn	ELISA	↑ Oligomeric α-Syn in PD vs. HC; correlated with UPDRS	Not reported	Not reported	Small cohort; limited clinical correlation
Daniele et al. 2018 [20]	54 PD / 48 HC	RBC	α-Syn/ Aβ heterocomplexes	ELISA	↑ α-Syn-Aβ complexes in PD; correlated with disease severity	Not reported	Not reported	Novel marker but requires replication
Papagianna	30 PD /	RBC	α-Syn dimers	ELISA	↑ α-Syn	Not	Not	Small sample;

Reference	Cohort (PD/HC)	Sample Type	Molecular Target	Assay Platform	Key Findings	Diagnostic Performance	Blinded Assessment	Limitations / Bias
kis et al. 2018 [21]	20 HC				dimerization in PD; no difference between genetic and sporadic	reported	reported	limited power for subgroup analysis

Note: This table provides detailed methodological information, diagnostic performance metrics, and quality assessments for studies cited in the main manuscript. AUC = area under the receiver operating characteristic curve; CI = confidence interval; ELISA = enzyme-linked immunosorbent assay; ECL = electrochemiluminescence; EV = extracellular vesicle; HC = healthy control; IMR = immunomagnetic reduction; MS = mass spectrometry; NDD = neurodegenerative disease; PD = Parkinson's disease; pS129 = phosphorylated serine 129; RBC = red blood cell; ROC = receiver operating characteristic; SAA = seed amplification assay; SIMOA = single-molecule array.

Supplementary Table S2. Meta-Analysis Summary of Peripheral α-Synuclein Levels in Parkinson's Disease Based on a comprehensive meta-analysis of studies published up to October 2022 [22].

Compartment / Fraction	Comparison	Effect Size (SMD)	95% CI	Sensitivity	Specificity	Quality of Evidence
Plasma α-Syn (total)	PD vs. HC	0.78	0.42–1.15	0.79 (0.64–0.89)	0.95 (0.90–0.98)	Moderate (high heterogeneity)
Plasma Neural-Derived Exosomal α-Syn	PD vs. HC	1.82	0.30–3.35	Not reported	Not reported	Low (limited studies)
Plasma Neural-Derived Exosomal α-Syn / Total α-Syn Ratio	PD vs. HC	1.26	0.19–2.33	Not reported	Not reported	Low
Erythrocytic α-Syn (total)	PD vs. HC	6.57	3.55–9.58	Not reported	Not reported	Moderate (high heterogeneity)
Serum α-Syn (total)	PD vs. HC	0.54	-0.27–1.34	Not reported	Not reported	Low (non-significant)
Saliva α-Syn	PD vs. HC	-0.85	-1.67 to -0.04	Not reported	Not reported	Low

Source: Adapted from meta-analysis by [9]. SMD = standardized mean difference; CI = confidence interval.

Interpretation: Plasma and erythrocytic α-Syn show the most consistent elevation in PD, with erythrocytic α-Syn demonstrating the largest effect size (SMD = 6.57), though this is accompanied by substantial heterogeneity across studies. Plasma α-Syn shows moderate discriminatory ability (sensitivity 79%, specificity 95%) but with considerable inter-study variability. Serum and saliva measurements are less consistent [9].

Supplementary Table S3. Analytical Performance of Detection Platforms for Blood α-Synuclein.

Parameter	ELISA	SIMOA	Mass Spectrometry (DIA/SWATH)	SAA (RT-QuIC/PMCA)
Limit of Detection	pg/mL – ng/mL	fg/mL – pg/mL	fmol – pmol	Amplification-based (depends on seed concentration)
Dynamic Range	~2–3 logs	~3–4 logs	~2–3 logs	Semi-quantitative (kinetic parameters)
Sample Volume Required	50–200 µL	25–100 µL	100–500 µL (with IP)	20–100 µL
Antibody Dependency	High (critical)	High (critical)	Low (peptide-level)	Low (substrate-dependent)
PTM Detection	Possible (antibody-dependent)	Possible (antibody-dependent)	Excellent (direct)	Indirect (seeding reflects conformation)
Throughput	High (96-well)	Moderate (96-well)	Low–Moderate	Moderate (96-well, multi-day)
Cost per Sample	$5–$20	$50–$150	$200–$500+	$50–$200
Turnaround Time	4–6 hours	3–5 hours	1–3 days	2–5 days
Inter-Laboratory CV	10–20%	5–15%	10–25%	15–40% (highly variable)
Clinical Readiness	Moderate (research use)	Low–Moderate (emerging)	Low (research use)	Low–Moderate (CSF approved; blood in development)

Abbreviations: CV, coefficient of variation; DIA, data-independent acquisition; ELISA, enzyme-linked immunosorbent assay; IP, immunoprecipitation; PMCA, protein misfolding cyclic amplification; PTM, post-translational modification; RT-QuIC, real-time quaking-induced conversion; SAA, seed amplification assay; SIMOA, single-molecule array; SWATH, sequential window acquisition of all theoretical fragment ion spectra.

Supplementary Table S4. CSF SAA Diagnostic Performance (Benchmark Reference).

Comparison	Sensitivity (95% CI)	Specificity (95% CI)	Study
PD vs. Healthy Controls	0.90 (0.82–0.95)	0.97 (0.82–1.00)	[23]
PD vs. MSA	0.81 (0.61–0.93)	0.97 (0.82–1.00)	[23]
Synucleinopathies vs. HC	0.88 (0.81–0.93)	0.97 (0.82–1.00)	[23]
Synucleinopathies vs. Tauopathies	0.88 (0.81–0.93)	0.67 (0.43–0.85)	[23]

Source: Data from αSyn-SAA studies [23]. Note that CSF SAA serves as the benchmark for blood-based assay development, though the lower specificity for distinguishing synucleinopathies from tauopathies highlights the challenge of differential diagnosis.

Notes on Supplementary Materials

These supplementary tables are designed to complement the main manuscript by providing:

1) Detailed study-level data (Table S1) that could not be included in the main Table 1 due to space constraints.

2) Meta-analytic summary (Table S2) of diagnostic performance across studies.

3) Comprehensive platform comparison (Table S3) with quantitative metrics.

4) Benchmark reference (Table S4) from CSF SAA for context.

The reported sensitivity and specificity values should be interpreted with caution due to significant inter-study heterogeneity in assay protocols, cohort characteristics, and pre-analytical variables. Performance metrics from single-center studies may not generalize to broader populations.

Supplementary References:

1. El-Agnaf, O. M. A., Detection of oligomeric forms of α-synuclein protein in human plasma as a potential biomarker for Parkinson's disease. *FASEB J.* **2006,** 20, 419–425.
2. Li, Q.-X., Plasma α-synuclein is decreased in subjects with Parkinson's disease. *Exp. Neurol.* **2007,** 204, 583–588.
3. Shi, M., Significance and confounders of peripheral DJ-1 and alpha-synuclein in Parkinson's disease. *Neurosci. Lett.* **2010,** 480, 78–82.
4. Duran, R., Plasma α-synuclein in patients with Parkinson's disease with and without treatment. *Mov. Disord.* **2010,** 25, 489–493.
5. Wang, L.; et al., A comparative study of plasma and erythrocytic α-synuclein in Parkinson's disease. *Neurodegener. Dis.* **2020,** 19, 204–210.
6. Zhao, H.-Q.; Li, F.; Wang, Z.; Wang, X.-M.; Feng, T., A comparative study of the amount of α-synuclein in ischemic stroke and Parkinson's disease. *Neurol. Sci.* **2016,** 37, 749–754.
7. Tian, C., Erythrocytic α-Synuclein as a potential biomarker for Parkinson's disease. *Transl. Neurodegener.* **2019,** 8, 15.

8. Li, X.-Y., Alterations of Erythrocytic Phosphorylated Alpha-Synuclein in Different Subtypes and Stages of Parkinson's Disease. *Front. Aging Neurosci.* **2021,** 13.
9. Liu, G.; Yu, Z.; Gao, L.; Zheng, Y.; Feng, T., Erythrocytic alpha-synuclein in early Parkinson's disease: A 3-year longitudinal study. *Parkinsonism Relat. Disord.* **2022,** 104, 44–48.
10. Yu, Z., Erythrocytic α-Synuclein Species for Parkinson's Disease Diagnosis and the Correlations With Clinical Characteristics. *Front. Aging Neurosci.* **2022,** 14, 827493.
11. Dimoula, K.; Papagiannakis, N.; Maniati, M.; Stefanis, L.; Emmanouilidou, E., Aggregated α-synuclein in erythrocytes as a potential biomarker for idiopathic Parkinson's Disease. *Parkinsonism Relat. Disord.* **2025,** 133.
12. Chang, C.-W.; Yang, S.-Y.; Yang, C.-C.; Chang, C.-W.; Wu, Y.-R., Plasma and Serum Alpha-Synuclein as a Biomarker of Diagnosis in Patients With Parkinson's Disease. *Front. Neurol.* **2020,** 10.
13. Ng, A. S. L.; Tan, Y. J.; Lu, Z.; Ng, E. Y. L.; Ng, S. Y. E.; Chia, N. S. Y.; Setiawan, F.; Xu, Z.; Tay, K. Y.; Prakash, K. M.; Au, W. L.; Tan, E. K.; Tan, L. C. S., Plasma alpha-synuclein detected by single molecule array is increased in PD. *Ann Clin Transl Neurol* **2019,** 6, (3), 615-619.
14. Foulds, P. G., Phosphorylated α-synuclein can be detected in blood plasma and is potentially a useful biomarker for Parkinson's disease. *FASEB J.* **2011,** 25, 4127–4137.
15. Cristiani, C. M.; et al., Erythrocytic α-synuclein in Parkinson's disease and progressive supranuclear palsy. *Biomedicines* **2024,** 12, 2510.
16. Kluge, A., Detecting Misfolded α-Synuclein in Blood Years before the Diagnosis of Parkinson's Disease. *Mov. Disord.* **2024,** 39, 1289–1299.
17. Okuzumi, A., Propagative α-synuclein seeds as serum biomarkers for synucleinopathies. *Nat. Med.* **2023,** 29, 1448–1455.
18. Schaeffer, E., Association of Misfolded α-Synuclein Derived from Neuronal Exosomes in Blood with Parkinson's Disease Diagnosis and Duration. *J. Park. Dis.* **2024,** 14, 667–679.
19. Wang, X.; Yu, S.; Li, F.; Feng, T., Detection of α-synuclein oligomers in red blood cells as a potential biomarker of Parkinson's disease. *Neurosci. Lett.* **2015,** 599, 115–119.
20. Daniele, S., α-Synuclein Heterocomplexes with β-Amyloid Are Increased in Red Blood Cells of Parkinson's Disease Patients and Correlate with Disease Severity. *Front. Mol. Neurosci.* **2018,** 11, 53.
21. Papagiannakis, N., Alpha-synuclein dimerization in erythrocytes of patients with genetic and non-genetic forms of Parkinson's Disease. *Neurosci. Lett.* **2018,** 672, 145–149.
22. Tang, Y.; et al., Alpha-synuclein in peripheral body fluid as a biomarker for Parkinson's disease: a meta-analysis. *Acta Neurol. Belg.* **2024,** 124, 831–842.
23. Fernandes Gomes, B.; Farris, C. M.; Ma, Y.; Concha-Marambio, L.; Nilsson, J.; Forsberg, K.; Lebovitz, R.; Andreasson, U.; Blennow, K.; Zetterberg, H.; Backstrom, D., Alzheimer's disease traits in Parkinson's disease without alpha-synuclein seeding. *Alzheimers Dement* **2025,** 21, (5), e70284.
